# SmartLaundry: A Real-Time System for Public Laundry Allocation in Smart Cities

**DOI:** 10.3390/s24072159

**Published:** 2024-03-28

**Authors:** Raluca Laura Portase, Ramona Tolas, Rodica Potolea

**Affiliations:** Computer Science Department, Technical University of Cluj Napoca, 400114 Cluj-Napoca, Romaniarodica.potolea@cs.utcluj.ro (R.P.)

**Keywords:** public laundries’ allocation, real-time system, forecasting usage, recommendation algorithm, IoT, smart city

## Abstract

Smart cities facilitate the comprehensive management and operation of urban data generated within a city, establishing the foundation for smart services and addressing diverse urban challenges. A smart system for public laundry management uses artificial intelligence-based solutions to solve the challenges of the inefficient utilization of public laundries, waiting times, overbooking or underutilization of machines, balancing of loads across machines, and implementation of energy-saving features. We propose SmartLaundry, a real-time system design for public laundry smart recommendations to better manage the loads across connected machines. Our system integrates the current status of the connected devices and data-driven forecasted usage to offer the end user connected via a mobile application a list of recommended machines that could be used. We forecast the daily usage of devices using traditional machine learning techniques and deep learning approaches, and we perform a comparative analysis of the results. As a proof of concept, we create a simulation of the interaction with our system.

## 1. Introduction

The global increase in population and urbanization has placed unprecedented pressure on cities worldwide. Projections indicate that 70% of the world’s population will reside in urban areas by 2050 [1]. Additionally, the escalating impacts of climate change and global warming underscore the imperative for these urban developments to prioritize sustainability and environmental responsibility, aligning with the United Nation’s Sustainable Development Goals for 2030 [2]. In response to these imperatives, smart cities have emerged as focal points for both governmental and private sector interests worldwide. Smart cities refer to an interlinked network of digital technologies, sensors, and data analytics. The purpose is to leverage technology to enhance the quality of life for urban residents while optimizing various aspects of urban functionality.

In the context of smart cities, multiple works have focused on providing solutions for better management of resources. Some examples are the resource allocation of charging stations [3], parking control [4], task scheduling in the cloud [5], or task scheduling for public laundry management [6]. Real-time applications stand as promising candidates for harnessing the computational capabilities offered by cloud computing environments, ensuring compliance with deadline constraints due to the fast-growing advancements in smart systems [7].

Nowadays, all devices are equipped with sensors to monitor their behavior and usage. One powerful way to extract actionable insights from these data is through time series prediction [8,9]. Using time series forecasting techniques to predict future usage patterns of devices has multiple applications, such as optimizing energy consumption, predicting equipment failures, or proactively allocating resources to enhance user experiences [10,11].

Public laundries have the disadvantage of inefficient machine usage. Some are overbooked, while others are underbooked. From the machine’s perspective, overuse leads to malfunctions and reduced operating time. From the user’s perspective, inefficient use leads to an increase in waiting time.

We propose a real-time system design for a smart city where public laundries have sensors attached to devices to monitor their functionality. Once recorded, usage patterns can be identified and extracted from functional data. Our system can be used for better resource management to optimize energy consumption and reduce peak demand by creating real-time recommendations regarding the advised washing centers for end users connected to an app via mobile devices. Once the smart recommendations are available, users can choose the one that best suits their particular needs, such as their current location or schedule. Moreover, our system would prevent inefficient utilization of the machines. To the best of the authors’ knowledge, a real-time system for an efficient recommendation of shared appliance allocation has not been previously studied in the literature. The system comprises three processing units, separated based on their purpose: data acquisition, a forecasting module, and a real-time recommendation module. The data-intensive operation, which consists of forecasting the next usage of devices using machine learning, is separated into its own module and uses the historical data stored in the cloud.

We summarize the main contributions of this paper as the following:

- A real-time system design for smart public laundry recommendation allocation.
- Data-driven forecasting for device usages.
- A resource allocation algorithm for smart washing center recommendations

Our system’s users need a response regarding the recommended devices at the moment they request them. Using our proposed solution presented in Section 3, which takes into account the machines’ current availability, the response is received in real time. We assume that all laundries have the same price. Our solution does not perform a weighted efficiency analysis on multiple criteria. We only take into account the devices’ real-time availability and the forecasted usage, which is based on their history.

The rest of the paper is structured as follows: Section 2 presents related work on smart city systems and time series forecasting. Section 3 presents our proposed system architecture and the algorithms used for smart washing machine recommendations. Section 4 compares results when using different forecasting techniques and a proof of concept for our system, and Section 5 concludes with some remarks.

## 2. Related Work

### 2.1. Smart City Solutions

Within the framework of smart cities, numerous endeavors have concentrated on delivering solutions to enhance resource management. Most works have focused on reducing energy consumption and mitigating the peak demand [12,13]. The total energy consumption at time *n* of a building with *m* appliances and *k* operating modes can be defined [14] as:

(1) $$energy\left(n\right)=\sum_{i=1}^{m}\left(\ldots \left(n\right),\ldots ,U_{k}^{i}\left(n\right)\right)\left(\begin{array}{c} \psi_{1}^{i}\\ \psi_{2}^{i}\\ \vdots \\ \psi_{k}^{i} \end{array}\right)+\varepsilon^{i}\left(n\right)$$

In this equation, *n* represents the moment of time, $U_{k}^{i}\left(n\right)$ is the operating On/Off status of the i-th appliance (which can be [0,1]), $\psi_{k}^{i}$ is the electricity consumed in a particular operating mode, and $\varepsilon^{i}\left(n\right)$ denotes the measurement of background noises. Lowering the peak demand of a system is important for ensuring a reliable and efficient supply of electricity while minimizing costs and environmental impacts. This can be achieved by better organizing the consumers connected to the network.

Smart city energy efficiency has been the topic of multiple works. A prediction method for future peak demand using deep learning architecture is suggested in [15]. Convolutional neural networks and long short-term memory are used to predict. In [16], a review of the work on generation, storage, infrastructure, facilities, and transport areas from the energy management and planning perspective is presented. Paper [17] presents the advances toward smart energy management in smart cities based on their application. The authors of this paper also present several case studies of cities that have already implemented smart energy management technologies.

The creation of virtual groups is an approach used to optimize collaboration and resource utilization within industrial settings. A smart collaborative evolvement scheme for virtual group creation in the context of a customized industrial Internet of Things is presented in [18]. A mapping graph was used to represent the group, which considered both physical and cyber distances to form the association index in edge weighting.

Several solutions are available in the literature for resource allocation problems. In [19], a three-layered network architecture composed of the IoT, edge, and cloud is proposed for smart cities. An auctionable mechanism is proposed to overcome the problem of allocating IoT-based edge resources. Specific techniques for distributing the computational workload to the network edge to self-computing nodes are explored in [20]. This can be particularly useful in a context characterized by limited resources and many restrictions that may be breached when performing analytical tasks. An adaptive multi-objective task scheduling algorithm is proposed in [5] for system resource scheduling on the hierarchical distributed cloud. This method is based on a particle swarm optimization algorithm [21].

A limited amount of work focuses on shared appliance allocation. The authors of [22] present the problem of laundry energy management system schedules and controls from the perspective of scheduling laundry tasks to maintain the monthly demand charge cost within a budget. In [6], a real-time rolling horizon-based optimization model for managing energy consumption in public laundries while considering users’ preferences is presented. The purpose of the model is to minimize energy usage, peak demand, and CO_2_ emissions. Neither one of these solutions focuses on load balancing between devices. Inefficient usage of shared appliances leads to errors in functioning. A better allocation of appliances would reduce the malfunctions. Therefore, even though it is not the objective of this work, our solution could be used for predictive and preventive maintenance. This could be performed by processing the initial intentions and forecasted usages in the context of preventive maintenance.

### 2.2. Time Series Forecasting

Time series data are the prevailing data type utilized in various domains [23]. The concept refers to a sequence of observations *x* recorded at specific time instances *t*, where *t* belongs to a predetermined set of times *T*.

One important application for time series modeling is forecasting. Time series forecasting relies only on historical data of a variable to predict future behavior. Various strategies exist for conducting forecasting. One of the most common ones is Vector Autoregression (with abbreviation VAR) [24], which has the disadvantage of not capturing nonlinearity patterns.

The random forest regression model can be used to predict multiple points in the future based on historical data by combining several single-point forecasts [25,26]. Extreme gradient boosting is a decision tree ensemble learning algorithm similar to the random forest that can be used for tasks such as classification and regression. It uses a gradient of the data for each tree, which makes the calculation faster and more accurate. XGBoost [27] implementation for an extreme gradient boosting method has also been successfully used for time series forecasting [28,29].

Deep learning methods have promising results for forecasting due to their ability to capture complex patterns and relationships in data. Temporal dependencies, nonlinear relationships, and high-dimensional time series data that are challenging for traditional statistical methods can be handled successfully by deep learning methods [30]. Convolutional Neural Networks (CNNs) were applied successfully on electricity price and load forecasting in [31]. Among the most successful approaches for recurrent neural networks is Long Short-Term Memory (LSTM). For time series predictions, LSTM tends to perform better than CNNs [32].

These methods do not perform well on data with high intermittency levels. The literature obtained better results after combining classical forecasting methods based on shared data characteristics, such as the intermittency level in [9], where the method was applied for forecasting the usage of household devices.

The evaluation metrics are essential to any machine learning linear regression or forecasting problem. Among the most commonly used metrics [33] is Mean Absolute Error (MAE), which represents the average variance between the actual and predicted values. Mean squared error (MSE) represents the average squared variance between the observed versus the predicted values. Another used metric is Mean Absolute Percentage Error (MAPE), which computes the forecast error as a percentage of the actual values. For regression problems where the output might be zero, percentage error metrics, such as MAPE [34], are not suitable; instead, Symmetric Mean Absolute Percentage Error (SMAPE) [35] can be used.

## 3. System Design

### 3.1. Proposed Terminology

When referring to data, we are going to use the following terminology:

- Raw real-time sensors’ data = raw data recorded by sensors attached to washing machines in a real-time manner.
- Preprocessed real-time data = data collected by sensors after the preprocessing step, which includes data selection and cleaning of the data.
- Historical data = the historical preprocessed data that were recorded and stored in the cloud system.
- Forecasted data = the forecasted usage data for the machines connected to the system.

Our system comprises three different processing modules based on their role in the system. We are further going to refer to them by using the terminology:

1. Data acquisition module = a module that receives the raw real-time data from sensors, extracts the information of interest, cleans it, and returns preprocessed real-time data.
2. Forecasting module = a module that predicts the usage on a given time interval per each device.
3. Real-time recommendation module = a module that communicates with the mobile device via an application. It uses preprocessed real-time data and forecasted data and sends recommendations regarding possible laundries.

### 3.2. SmartLaundry—Real-Time System Architecture

Our proposed system uses raw data collected by sensors attached to the washing machines from public washing centers to create real-time smart recommendations regarding the available machines and public laundries. We use a data-driven approach to forecast the usage of machines. The recommendations are based on real-time data and are available to mobile devices connected to the system using a mobile application and the forecasted load of the centers. Once the recommendations for available devices are received, the user can choose the one that best suits their schedule, geographic location, and available resources.

**Figure 1:**
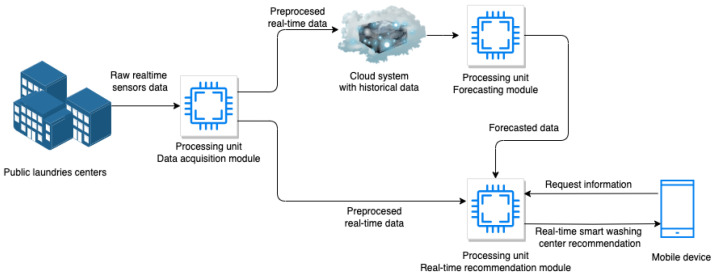
High-level design of SmartLaundry—the real-time system for managing public laundries.

Figure 1 illustrates our proposed real-time system architecture from a high-level perspective. The sensors attached to the washing machines record the functioning data of the devices in real time. Once recorded, data are sent to the data acquisition module and preprocessed. The information extracted is stored in the cloud system, which contains the historical usage data from all the connected devices. By using this approach, the data-intensive load is moved to the cloud. Based on historical data, the forecasting module predicts future usage. The real-time recommendation module uses forecasted data regarding the usage of the devices and their current running status and will compute a list of recommended available devices to be used. The system users interact with it by using an application on their mobile phones. This way, not only will the waiting time be reduced, but resources will be allocated so that overbooking or underutilization of the machines will be avoided.

**Data acquisition module.** IoT data coming from devices are considered big data due to their characteristics, such as volume, variety, and velocity. Therefore, the first appropriate step in the data acquisition module is to extract the usage data of interest to overcome the dataset dimensionality complexity. The next step is the semantical and syntactical cleaning of the raw data. A comprehensive and versatile methodology tailored for data analysis and cleaning was introduced in [36].

Our system’s devices are designed to function in cycles. One cycle is defined by the beginning of a task, its running (with the purpose of solving the task), and its stopping. For example, in the case of washing machines, the task is to clean the clothes. In [37], a method for detecting cycles from raw data recorded from devices with running patterns similar to the ones described above is introduced.

Based on the status of the devices, if the beginning of a cycle is detected, the running status information is updated to notify that the machine is currently busy. If the end of a cycle is detected, the current run time of the device is updated in the cloud, and the information regarding the new status of the device is sent.

**Figure 2:**
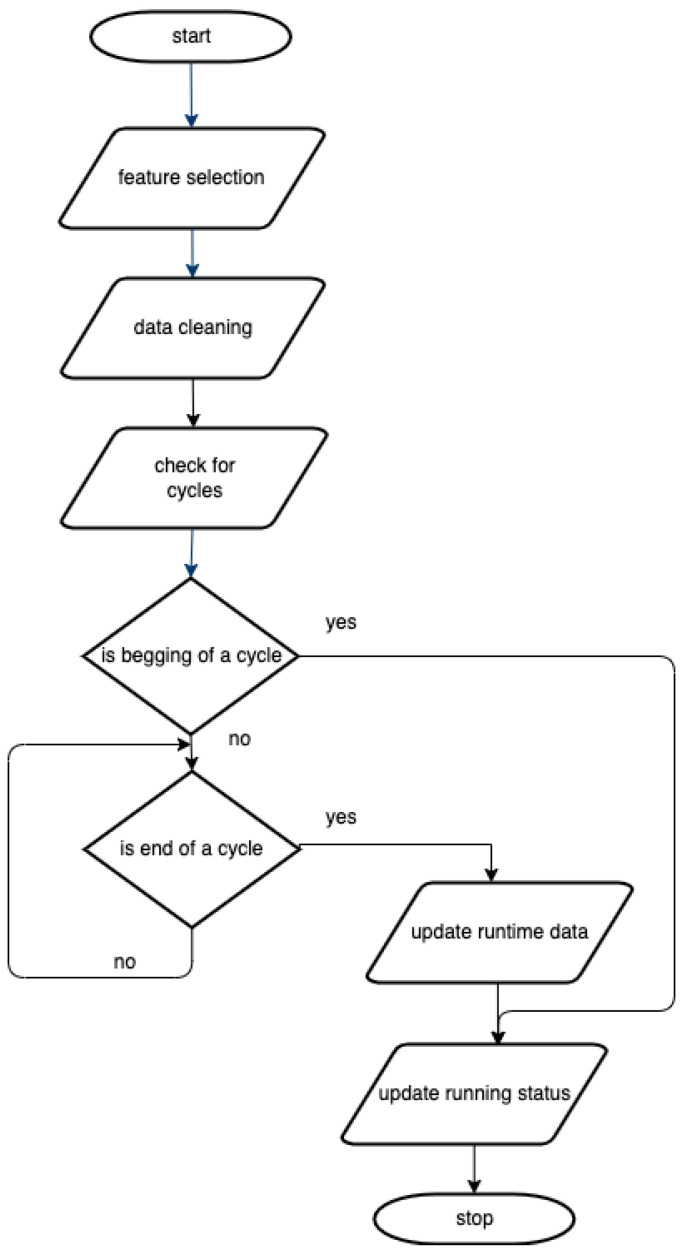
Data acquisition module flow diagram.

The flow diagram of the data acquisition module is presented in Figure 2.

**Forecasting module.** The forecasting module uses historical data stored in the cloud system to forecast the usage of washing devices. We are allocating cloud computing resources to the forecasting task so that the overall timing constraints of the smart devices remain intact. The forecasting module returns a list of the predicted values per device for a given period of time. Based on the predicted usage, the devices with the highest demand are identified and, therefore, not considered for the suggested list of recommended devices in case enough devices are available.

The granularity level refers to the time interval for which we are performing the prediction. For example, a granularity of a day means the system will predict the daily usage of the devices. This implies that the forecasting task should be a periodic task that runs daily. Smaller granularities, such as a couple of hours, could be used for a more adequate prediction. Our system can be easily configured to select the granularity of the prediction.

Our problem is similar to task scheduling, which is an NP-hard problem. Data-driven approaches offer better solutions when qualitative and quantitative data are available. Machine learning models can outperform traditional statistical methods and are well-suited for handling large volumes of data. Therefore, our proposed method uses machine learning algorithms for forecasting.

**Real-time recommendation module.** The purpose of selection is to reduce the usage of the devices that are used the most so that resources can be allocated better. The real-time recommendation module uses the devices’ real-time data status and forecasted usage to suggest a list of possible laundries to be used.

This module uses two different customizable parameters:

- AllowedThreshold represents the maximum load a machine allows. It is a parameter set depending on the number of machines and overall system usage.
- MinListLength represents the targeted minimum number of machines recommended for the user of the system at a given moment of time. The user should select it according to the possibility of movement and could incorporate the distance from a suggested location (not yet incorporated).

This module uses the implementation of two algorithms presented in the next session to select the suggested machines list. Only the running status of devices is used in this module to minimize the real-time module’s computational cost. The forecasting data are already computed on the previous layer.

### 3.3. Algorithmic Method for Smart Real-Time Recommendations of Machines

Algorithm 1 illustrates the construction of the list with the devices for which we want to reduce the usage. A better load balancing between machines can be achieved by implementing a penalization method and recommending the devices that tend to be overutilized. The algorithm’s output is a list ordered ascending based on the forecasted usage data. Only the devices that, according to forecasting, will be used over a given threshold will be included in this list.
**Algorithm 1:** GetPenalizedDevices: Determination of the list of the devices with high forecasted usage.
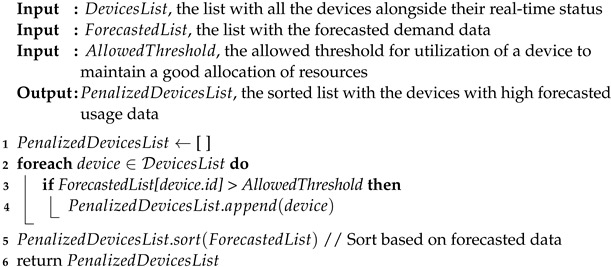


The output list of the algorithm is initially empty in step 1. The algorithm takes all the devices from the system. In steps corresponding to lines 3 and 4 of the algorithm, the output list is constructed only by those devices with the forecasted value over the given AllowedThreshold parameter. In order to reduce the load of the most used forecasted one, the list is sorted in ascending manner based on the forecasted values of the devices in step 5. Therefore, the devices that are forecasted to be used the most will be suggested the least and will appear in the suggestion list only if there are not enough devices available.

Algorithm 2 is used to construct and return the recommendation list of the devices to be used. At the beginning of the algorithm, the output list is initialized with the empty list. In line 2, our proposed algorithm takes the list of the devices with high forecasted usage by calling Algorithm 1. Further on, it creates a list of recommended devices from all the devices that are not currently running and are not on the penalization list. The running status information data come from the preprocessed real-time data (output of the data acquisition module).

The algorithm aims to create a list of lengths of at least MinListLength of the best-suited machines. After step 5, the list will be returned if we have already achieved the goal. Otherwise, in steps 7–10, the algorithm will add devices that are not currently running from the penalization list (output of Algorithm 1). If we achieve the needed device list length or there are no more devices in the penalization list, we will end the algorithm and return the recommended devices list.

Algorithm 2 uses the current time for the suggested list. If a list of potentially free devices in a time interval Δt is preferred, a slight modification of Algorithm 2 is required. A new parameter timeLimit should be added, representing the time the system user would arrive at the possible locations. At this point, the information about the device’s current running status is insufficient to determine if the device will still be busy after the timeLimit time. To overcome this, the expected number of minutes in which the machine will finish its running cycle must be computed in the data acquisition module. The running status conditions from lines 4 and 9 from Algorithm 2 should be changed from device.status!=RUNNING to device.status!=RUNNING *or* device.expectedFinishTime<timeLimit.

By updating the recommended list based on the current status of the devices and by adding last to the list OF the devices that are forecasted to be used the most (only if there are not enough available devices with a small predicted load), the overall load of the most used centers will be reduced. Moreover, using our proposed algorithm leads to a reduction in the waiting time because only the free devices will be visible.
**Algorithm 2:** GetRecommendedDevices: Determination of the list of the recommended devices to be used.
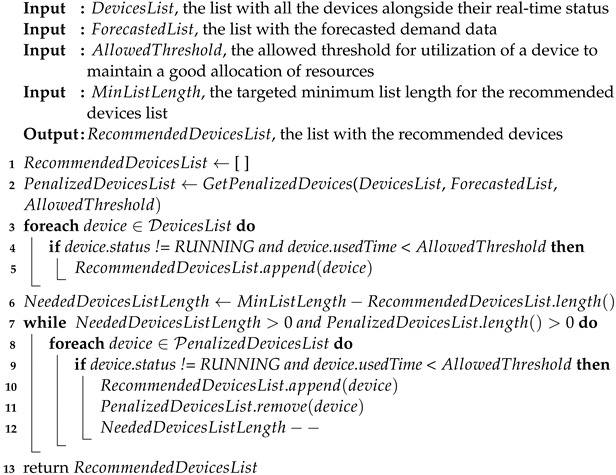


## 4. Proof of Concept Evaluation

### 4.1. Data Acquisition and Forecasting on Real Devices’ Data

We used machine learning algorithms for forecasting and compared the results of several methods for forecasting the future utilization of appliances from real data recorded from past utilization. For our experiments, we used an initial raw sensors dataset consisting of 8.1 million washing machine devices recorded during one year.

**Figure 3:**
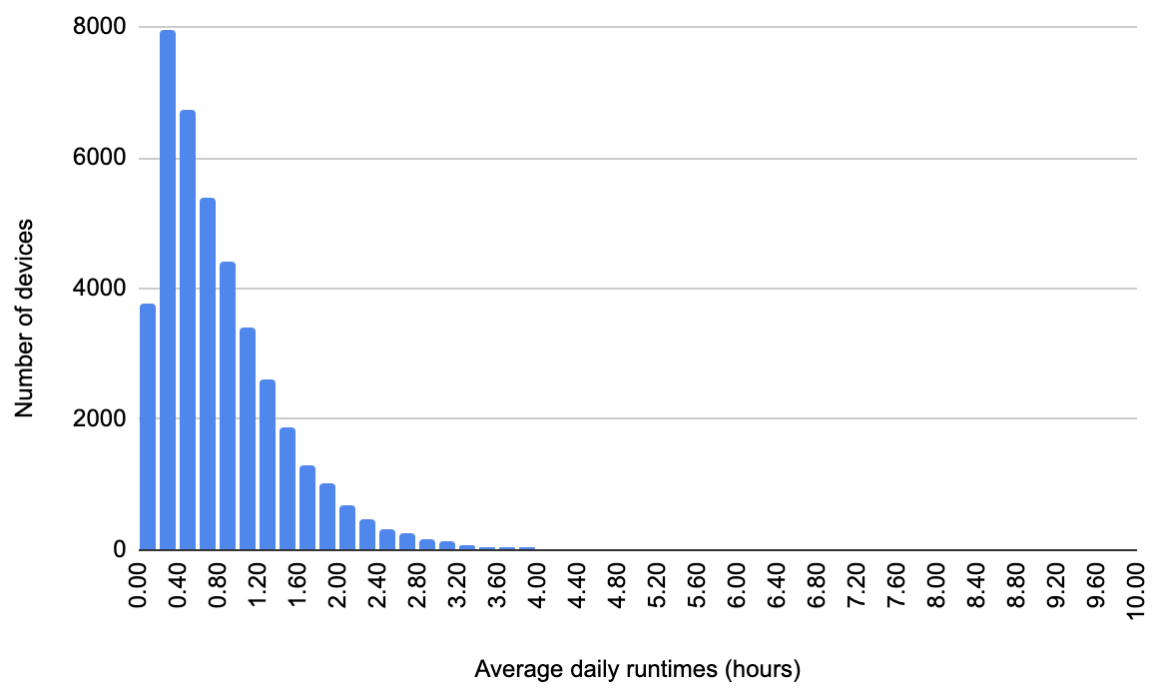
Histogram of the number of appliances and their daily average run time over a year.

Figure 3 presents the usage patterns of all the appliances from the initial raw sensors dataset. The histogram contains the average number of hours an appliance was used per day. The maximum average daily amount a device was used in our dataset is 10 h, representing the upper limit on the horizontal axis. On the vertical axis, we have the number of devices. As can be seen, most of the devices are used for under 3 h. Due to the differences in the number of devices with average run time in the interval [3 h; 10 h], some devices are not visible in the histogram.

After computing the daily running time of the devices, we removed from the initial raw dataset the devices that have an average run time of less than 3 h per day. The mean run time values include the weekends and legal holidays. The dimensions of the initial dataset compared to the one after selecting instances are presented in Table 1.

**Figure 4:**
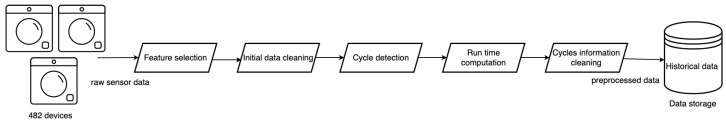
Steps followed to transform raw data from sensors to historical data.

On the selected raw sensors dataset, we applied the steps corresponding to the data acquisition module illustrated in Figure 2 to select only the features corresponding to the running of the devices. We cleaned the data and detected the run cycles, marked their start and end, and computed the overall running duration for each cycle. We eliminated the washing cycles that lasted less than 5 min or more than 5 h for a supplementary cleaning step and created our historical dataset from the resulting data. Historical data were further used in the forecasting module. Figure 4 illustrates the steps performed to transform the raw sensor data into historical data that were used for forecasting.

For the evaluations, we selected several different appliances. Among the investigated ones, we present the results for five appliances representative of their categories out of the 482 devices. Their characteristics are summarized in Table 2, which we will further refer to. All of these appliances have different numbers of days without usage per year and different average run times per day.

Several regression models have been successfully used to predict future values for time series data. Due to their results from related works, we selected random forest, XGBoost, and SVR. Methods such as VAR were not selected due to the disadvantage of not capturing nonlinearity patterns. Therefore, we implemented and compared the results for daily prediction by using random forest, XGBoost, and SVR. In our experiments, we used scikit-learn library [38] for implementation of the regression algorithms. We used 11-month data for training and predicted the devices’ usage for a month’s window size.

From the metrics available in the literature for evaluating forecasting, we selected Mean Absolute Error (MAE)

(2) $$MAE=\sum_{i=0}^{N-1}\left|x_{i}-y_{i}\right|$$

Furthermore, we used Symmetric Mean Absolute Percentage Error (SMAPE), which we normalized at the [0,100] interval.

(3) $$SMAPE=\frac{100\%}{N}\sum_{i=0}^{N-1}\frac{2*(x_{i}-y_{i})}{x+y}.$$

In our equations, *x* represents the predicted values, *y* is the actual values, and *N* is the number of observations.

There are days when laundry centers are closed, such as public holidays. Therefore, the run time series will have entries with values equal to 0, which means the devices were not used. MAPE as a metric is unsuitable for data with 0 values, so we did not use it in our experiments. The results of our forecasting experiments are presented in Table 3.

Since MAE represents the mean error, a smaller value illustrates a more accurate prediction. A SMAPE value of 0 percent signifies a perfect alignment between the actual and the forecasted values. A value of 100% implies no similarity between the predicted and actual values. In Table 3, we use bold text to suggest the best-obtained results. Our results for the best configurations have values for SMAPE between 8.69% and 20.89% for all the devices illustrated in the experiments. From the perspective of the forecasting methods, XGBoost performed best, with random forest as a close contender. SVR performed worse for daily usage prediction.

Deep learning methods have successfully been used to forecast time series. Among the methods with promising results in the literature are CNNs and LSTM. We applied these methods to forecast our devices’ daily usage and illustrate the results in Table 4. We used the same metrics as above (MAE and SMAPE), and we predicted the usage based on 11-month data for a window size of a month. The best configuration identified for each of the architectures was used. For LSTM, we used Adam optimizer and 200 neurons. For CNNs, we used 64 filters, kernel size 5, activation relu, and Adam optimizer.

We use bold text to suggest the best-obtained results. From the MAE perspective, LSTM consistently achieved better results than CNNs. In most cases, we obtained better SMAPE results with LSTM. Comparing the results of the traditional methods from Table 3 with the ones obtained with deep learning methods, deep learning methods outperform the traditional methods from the perspective of mean average value.

### 4.2. Simulation of the Interaction with the Recommendation System

**Figure 5:**
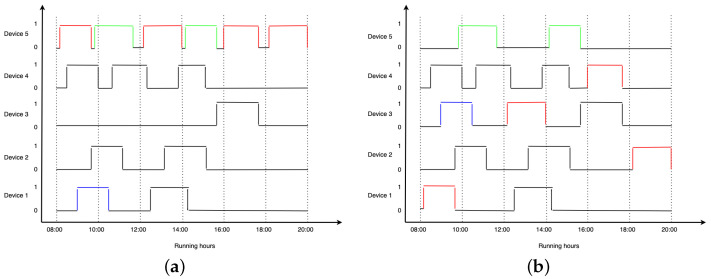
Simulation view of the allocation algorithm applied for 5 devices. The cycles marked with red represents the cycles that are moved to another machine; Green represents the cycles from the highly used Device 5 that remain on the same machine; Blue represents the cycles that had to be reallocated due to machine being busy with another task (**a**) Simulation view of the initial allocation of washing tasks. (**b**) Simulation view of the allocation of washing tasks after our proposed algorithm is used.

To illustrate our proposed algorithm for smart real-time machine recommendations, a simulation of the interaction has been made. We consider the machines as being localized at different locations and operational between hours 8:00 and 20:00, with a variable duration of cycles. To maintain the visual explainability of the system, five devices have been chosen, where four of them are forecasted to be used below the AllowedThreshold, and one of them over it. For MinListLength, we use a value equal to 3. Figure 5a illustrates the forecasted running cycles of the simulated machines. On the OX axis, we have the running hours of the devices, and on the OY axis, we have the state of the devices from 1 to 5. When the value for each device is 0, it means that the device is not running, while a value of 1 means it is running.

Figure 5b illustrates a possible allocation of the washing tasks after our proposed algorithm is used. Due to the fact that the user can choose one of the available machines, the simulation result is not unique. Since device 5 is used over the selected threshold, it means it will be in the penalization list.

At hour 8:00, the list of recommended devices to be used will contain only devices from 1 to 3. We consider that the user will select device 1 for the first washing cycle (the first one marked with red) instead of device 5. Consequently, when the user corresponding to the initial first task from device 1 (marked with blue) uses the system, device 1 will appear as unavailable, and the user will choose device 3 for the task.

Slightly before 10:00, another user tries to use the system to wash their clothing, a cycle marked with green. At this point, devices 1 to 4 will be busy, so in the recommendation list, only the penalized device 5 will appear available so that the task will remain on device 5. After 12:00, a new user uses the system to perform the second red cycle. At this point, devices 1, 2, 3, and 5 are available. Since the length of our recommended list is equal to 3, device 5 will not appear as recommended. Therefore, the user will use another device from the list—in this case, device 3.

For the next task of device 5, devices 1, 2, and 4 will be busy, so the list of available devices will contain devices 3 and 5. Since device 5 is available, the user will proceed as initially planned, and the green task will remain on the originally chosen device. For the last two tasks of device 5, there will be at least three other devices available, so the user will choose another device for their washing task.

Figure 5 compares the scenario without the proposed solution—Figure 5a, and the same scenario after our system is applied—Figure 5b. Initially, device 5 was overutilized—with six tasks, while device 3 was underutilized, with just one task assigned to it. As a result of this simulation, all our devices are running between two and four tasks. The better load management between washing tasks illustrates the significance of this work.

## 5. Conclusions

In the context of smart cities, public laundries are connected to the internet, and users can see the real-time status of the devices for convenient yet energy-efficient resource management. We presented a design for a real-time system that can be used to better balance loads across machines. Our design is composed of three processing units: a data acquisition module, a forecasting module, and a real-time recommendation module. Users interact with the system using a mobile application to see real-time data.

We provided a data-driven approach for forecasting device utilization based on data logs recorded by sensors attached to devices that record their functionality. We proposed an algorithm for selecting a list of suitable machines from all the public laundry centers to prevent their inefficient utilization. The algorithm uses the real-time status of the devices to see which devices are currently available and forecast data for better load balancing across machines.

We used both traditional machine learning algorithms and a deep learning approach for forecasting and proved the possibility of taking advantage of these data by comparing the results of several methods for forecasting the future utilization of appliances from real data recorded from past utilization. We created a simulation of the interaction with our system as a proof of concept.

## Figures and Tables

**Table 1:** Dimensions of the initial dataset versus selected and cleaned instances over one year.

Dataset	Size	No of Devices
Initial raw sensors dataset	8.1 millions	49 k
Selected raw sensors dataset	530 k	482

**Table 2:** Numerical characteristics of appliances used for experiments.

Appliance	Average Usage/Day (s)	No of Days without Usage/Year
App 1	36.718	39
App 2	31.492	38
App 3	21.159	48
App 4	23.916	39
App 5	21.475	50

**Table 3:** Results of daily usage forecasting for washing machines after using three different traditional methods.

	Random Forest		XGBoost		SVR	
Metrics	MAE	SMAPE	MAE	SMAPE	MAE	SMAPE
App 1	**9119**	**12.14%**	**9119**	**12.14%**	14,539	36.51%
App 2	**6045**	**9.66%**	6075	9.76%	9669	28.67%
App 3	4042	22.02%	**4012**	**8.69%**	6153	13.81%
App 4	**10,198**	**15.37%**	**10,198**	**15.37%**	15,048	37.42%
App 5	8522	29.22%	**8432**	**20.89%**	9728	40.34%

**Table 4:** Results of daily usage forecasting for washing machines after using deep learning methods.

	CNN		LSTM	
Metrics	MAE	SMAPE	MAE	SMAPE
App 1	8168	**12.37%**	**6659**	20.86%
App 2	4423	6.43%	**4311**	**6.34%**
App 3	4428	22.41%	**4348**	**9.63%**
App 4	11,466	19.77%	**11,021**	**19.41%**
App 5	5956	26.61%	**4846**	**26.22%**

## Data Availability

The authors do not have permission to disclose the data.
